# Low-energy and tunable LIF neuron using SiGe bandgap-engineered resistive switching transistor

**DOI:** 10.1186/s11671-024-04079-5

**Published:** 2024-08-23

**Authors:** Yijoon Kim, Hyangwoo Kim, Kyounghwan Oh, Ju Hong Park, Byoung Don Kong, Chang-Ki Baek

**Affiliations:** 1 Department of Convergence IT Engineering, Pohang University of Science and Technology (POSTECH), Pohang, 37673 South Korea; 2 Future IT Innovation Laboratory, Pohang University of Science and Technology (POSTECH), Pohang, 37673 South Korea; 3 Department of Electrical Engineering, Pohang University of Science and Technology (POSTECH), Pohang, 37673 South Korea

**Keywords:** Spiking neural networks (SNNs), Leaky integrate-and-fire (LIF), Tunable function, Bandgap engineering

## Abstract

We have proposed leaky integrate-and-fire (LIF) neuron having low-energy consumption and tunable functionality without external circuit components. Our LIF neuron has a simple configuration consisting of only three components: one bandgap-engineered resistive switching transistor (BE-RST), one capacitor, and one resistor. Here, the crucial point is that BE-RST with a silicon–germanium heterojunction possesses an amplified hysteric current switching with a low latch-up voltage due to improved hole storage capability and impact ionization coefficient. Therefore, the proposed neuron utilizing BE-RST requires an energy consumption of 0.36 pJ/spike, which is approximately six times lower than 2.08 pJ/spike of pure silicon-RST based neuron. In addition, the spiking properties can be tuned by modulating the leakage rate and threshold through gate bias, which contributes to energy-efficient sparse-activity and high learning accuracy. As a result, our proposed neuron can be a promising candidate for executing various spiking neural network applications.

## Introduction

Spiking neural networks (SNNs) demonstrate significant energy efficiency by mimicking the biological nervous system that process binary spike signals in a parallel and event-driven manner [1–3]. Spike events are typically generated based on the leaky integrate-and-fire (LIF) neuron model [4, 5]. It is crucial that a substantial number of LIF neurons are required to solve parallel and complex tasks such as classification and recognition. In other words, the design of compact and low-energy LIF neurons is required to realize space- and energy-efficient SNN hardware. However, many of the reported LIF neurons were not sufficient to meet these requirements. Complementary metal oxide semiconductor (CMOS)-based neuron circuits require many transistors and capacitors, which results in substantial space occupancy and energy consumption [6–8]. To address these critical issues, partially depleted (PD) SOI-MOSFET, L-shaped gate bipolar impact ionization MOS (L-BIMOS) and double gate-junctionless field effect transistor (DG-JLFET) have been replaced the leaky-integration function by accumulating charge in the floating-body instead of a capacitor [9–11]. However, the absence of an automatic reset function necessitates a reset circuit for periodic LIF operation and current-to-voltage signal converters are required to ensure stable compatibility with synaptic device arrays. The operation of these external circuits inevitably leads to significant energy consumption of ~ 0.3 nJ due to the additional voltage supply requirement [9, 12]. In another proposal, *biristor*, SOI-MOSFET and germanium (Ge)-MOSFET have been suggested as a single-device neuron by using the single transistor latch phenomenon [13–16]. However, the drawback lies in the significant energy consumption of ~ 0.95 nJ resulting from wide spike pulses and large latch-up voltages.

Alternatively, LIF neurons based on volatile threshold switching (TS) devices can overcome these limitations due to their low energy consumption and compact circuit configuration [17–21]. The TS device is a type of volatile memristor that exhibits hysteric-switching behavior through electronic, atomic, and thermal phenomenon [22–24]. These TS device neurons consume less energy due to the low operating voltage range and fast switching speed of TS devices. Additionally, the hysteresis properties of the TS device enable periodic neural oscillations without the large external circuitry which consumes additional energy. However, TS devices face challenges in practical applications because their characteristics are difficult to control consistently in non-CMOS compatible large-area fabrication. Most importantly, most TS devices applied to LIF neurons have gateless structures, making it impossible to control their spiking characteristics without hardware modifications. The tunable threshold functionalities are essential at the neuron level as they can further improve learning accuracy and energy efficiency by filtering input signals within a certain range and preventing excessive firing [25–27]. This tunable feature can also be applied to fuzzy tasks by fine-tuning the cluster's threshold, which allows the system to more accurately classify and respond to data with varying degrees of ambiguity [28–30]. Moreover, data loss can be averted by adjusting the spiking frequency to maintain stable operation, even with wide variations in conductance of synaptic devices [15, 31, 32]. The three terminal TS device is applied to LIF neuron for tunable function [21]. However, this neuron generates very high spike voltage and has a narrow range of adjustable spiking frequency.

A resistive switching transistor (RST) is a three-terminal transistor that exhibits volatile resistive switching characteristics based on the impact ionization effect. The RST which can be compatible of CMOS fabrication has a simple nanowire structure consisting of physical N-P-N silicon (Si) layers. Additionally, the gate structure can implement a wide range of tunable spike functions. However, during spike firing, a lot of energy is consumed due to the high latch-up voltage and slow switching speed.

In this study, we propose a CMOS compatible LIF neuron using a gated silicon–germanium (SiGe) bandgap engineered RST (BE-RST) for low energy and tunable applications through TCAD calibrated simulation. Periodic neural oscillation is successfully achieved without external circuit by using the hysteresis characteristics of the BE-RST. To emphasize the advantages of our proposed neuron, the energy consumption is compared to a LIF neuron, where the pure Si-RST is used as a replacement. The LIF operation can be intentionally tuned through the modulation of the off-state resistance and latch-up voltage of the hysteresis characteristics. With these adjustable characteristics, an analysis is conducted in terms of frequency, peak-to-peak voltage and energy consumption in response to variations in gate voltage.

## Results and discussion

### Structure & characteristics of LIF neuron

**Fig. 1:**
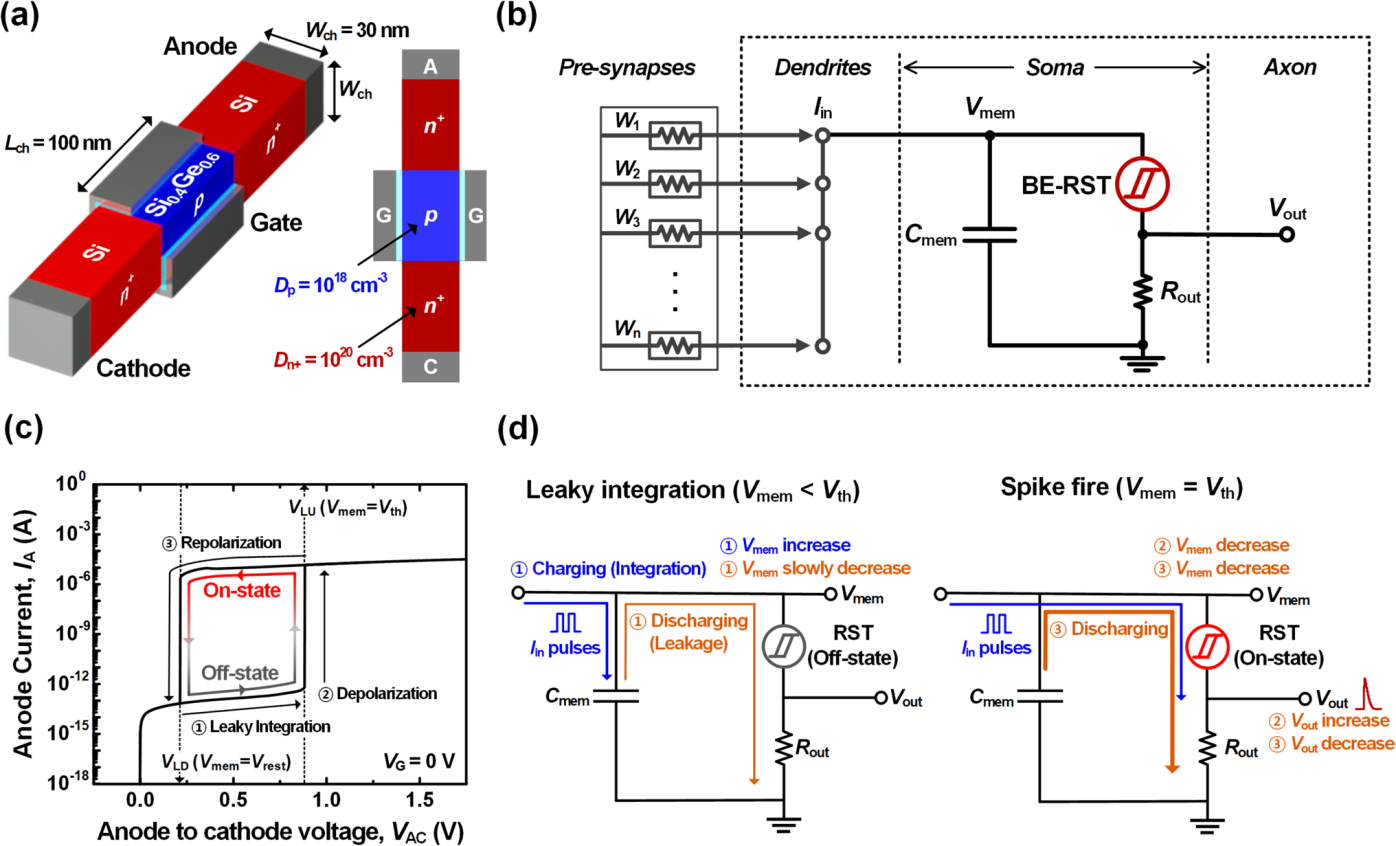
BE-RST based LIF neuron **a** 2D cross-sectional view and 3D schematic diagram of the BE-RST, **b** circuit diagram of LIF neuron which similarly implemented the functions of dendrite, soma and axon in biological neuron, **c** *I*_A_-*V*_AC_ hysteresis characteristics of the BE-RST at *V*_G_ = 0 V and **d** flowchart of LIF operation: leaky-integration and spike generation

Figure 1a shows the 2D cross-sectional view and 3D schematic diagram of proposed BE-RST. The BE-RST features a Si nanowire-structure consisting of *n*^+^-anode–*p*-channel–*n*^+^-cathode layers with the *p*-channel exceptionally made of SiGe material. The Ge content (*x* in Si_1-*x*_Ge_*x*_) is set to 0.6 to ensure the narrow bandgap for the *p*-channel. The channel area (*W*_ch_^2^) of the nanowire is designed to be 30 × 30 nm^2^ to minimize defects caused by lattice mismatch between Si and SiGe, considering the critical thickness to Ge content [33]. This nanowire with SiGe hetero-bandgap structure can be fabricated via a condensation technique that concentrates Ge during a high-temperature gate oxidation process [34]. The anode and cathode are highly doped with *D*_n+_ of 10^20^ cm^−3^. Considering sufficient impact ionization effect and hysteresis margin, the doping concentration (*D*_p_) and length (*L*_ch_) of *p*-channel are 10^18^ cm^−3^ and 100 nm, respectively. Figure 1b depicts the basic unit block of neuromorphic hardware, consisting of interconnected pre-synapses and BE-RST based LIF neuron. The pre-synapses transmit current signals proportional to its intrinsic weights (*W*_1_, *W*_2_,…, *W*_n_) to the LIF neuron. Our proposed LIF neuron, shown in the dotted box, consists of three functional parts similar to a biological neuron: input electrical cables (dendrites), a cell body consisting of the BE-RST, a capacitor, and a resistor (soma), along with output electrical cables (axon). In particular, the pivotal functional part of the neuron is the cell body, which integrates synaptic inputs into the membrane potential and generates spiking events when the threshold is reached [35]. The capacitor (*C*_mem_ = 1 pF) integrates the weighted input current signals (*I*_in_) in the form of a membrane potential (*V*_mem_). The *C*_mem_ was set to the minimum value that prevents overflow or underflow from pre-synaptic array, taking into account the *I*_in_ level [36]. The BE-RST plays a role in triggering a spike when the *V*_mem_ reaches the threshold potential (*V*_th_), resembling ionic channels embedded inside the membrane of soma. The resistor (*R*_out_ = 30 kΩ) in series with BE-RST divides the *V*_mem_ to generate an output spike voltage (*V*_out_). Figure 1c shows anode current (*I*_A_)-anode to cathode voltage (*V*_AC_) hysteresis characteristics of BE-RST when the gate voltage (*V*_G_) is 0 V. The BE-RST rapidly transitions to the on-state at the latch-up voltage (*V*_LU_) through the positive-feedback mechanism based on the impact-ionization effect. Afterward, during reverse *V*_AC_ sweeping, the BE-RST remains on-state and returns to the off-state at the latch-down voltage (*V*_LD_). By this mechanism, the proposed circuit configuration utilizing RST device can accurately reproduce leaky integration, depolarization, and repolarization of biological neurons. Figure 1d illustrates flow-chart of LIF operation, which consists of a leaky integration step and a spike fire step. When the *I*_in_ signal is input, the RST is in the off-state, so the *C*_mem_ is predominantly charged, increasing *V*_mem_. This increment corresponds to excitatory post-synaptic potential resulting from synaptic integration. On the other hand, the *V*_mem_ decreases slowly during the interval time (*t*_int_) as the *C*_mem_ discharges slightly through the RST. This decrease reflects to the leaky behavior of the biological neuron. When the accumulated *I*_in_ causes the voltage to reach RST (*V*_mem_–*V*_out_) to the *V*_LU_ where the *V*_mem_ aligns with the *V*_th_, a sharp decrease in the resistance of RST increases the *V*_out_. This increase in the *V*_out_ corresponds to the depolarization. At the same time, the *I*_in_ flows through the low-resistance RST in the same direction of discharging path. Then, the *C*_mem_ begins to discharge without any further charging, reducing the *V*_mem_. The *V*_out_ decreases proportionally to the *V*_mem_, leading to the repolarization. During this period, no further firing can occur from the input of additional *I*_in_ because the low-resistance RST prevents the *C*_mem_ from being charged. This time corresponds to the refractory period. When the voltage across RST reaches the *V*_LD_, the RST quickly returns to the off-state, at which point *V*_mem_ matches the resting potential (*V*_rest_). This self-reset process completes the spike generation and automatically prepares the leaky integration operation for the next spike event. Therefore, periodic neural oscillation can be implemented through the counter-clockwise hysteresis of the RST.

**Fig. 2:**
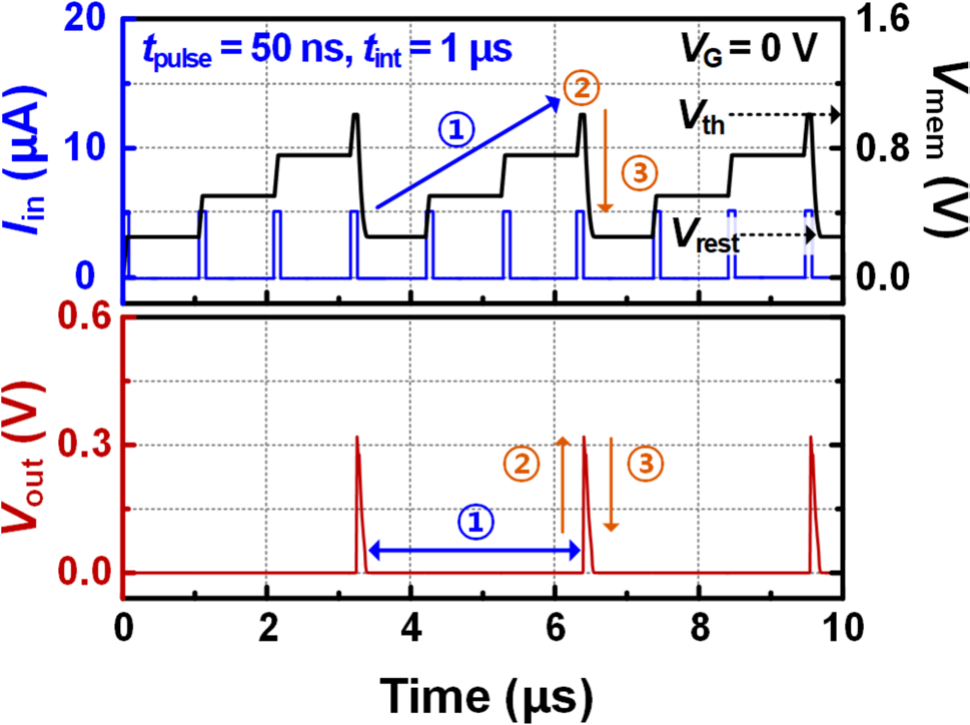
Spike response in time domain when *I*_in_ pulses of 5 μA are input

**Fig. 3:**
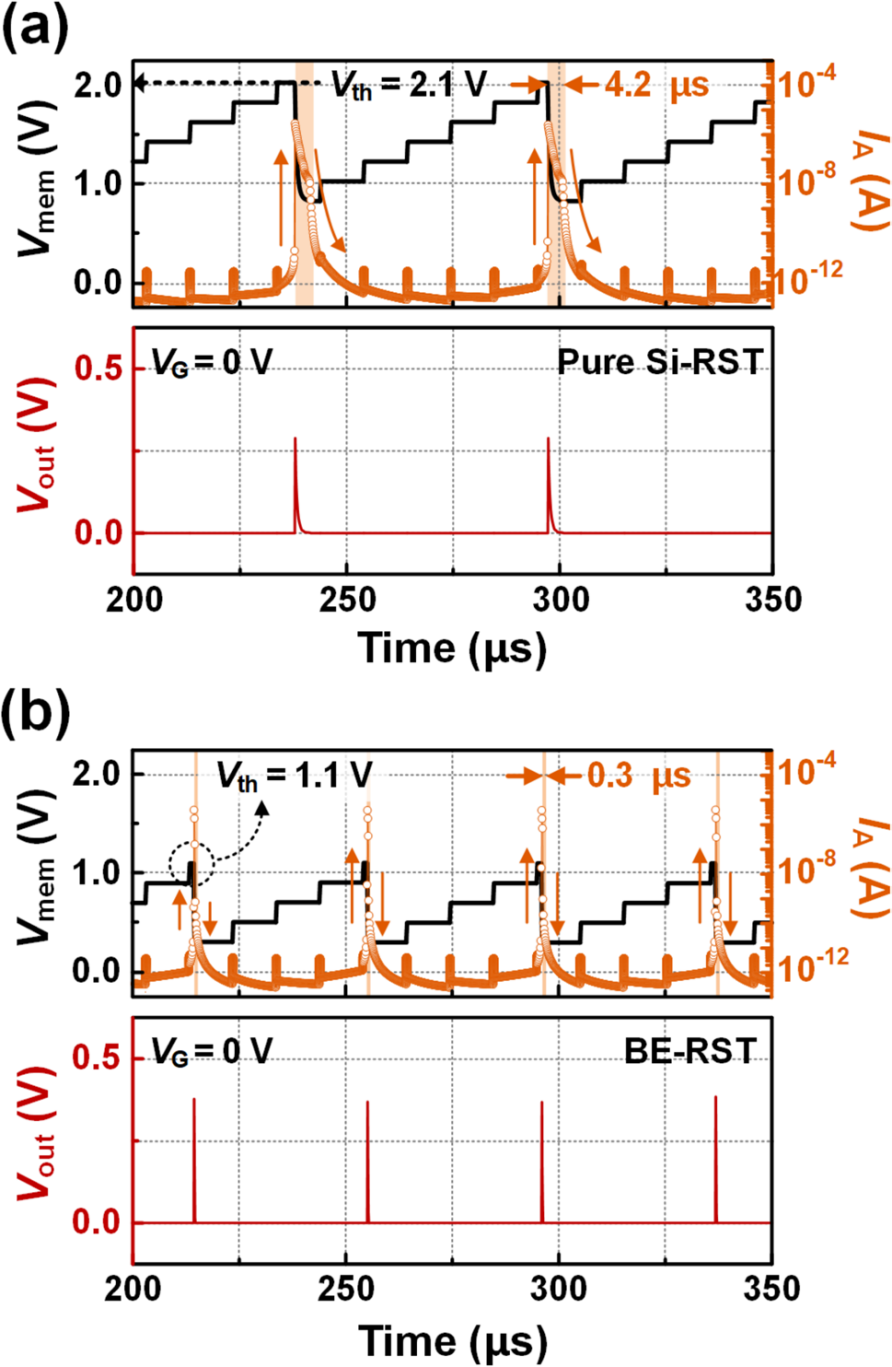
Comparison between **a** pure Si-RST and **b** BE-RST based LIF neuron in terms of *I*_A_, *V*_mem_ and *V*_out_ as a function of time

Figure 2 shows the periodic spike response of the *V*_out_ based on the *I*_in_, and *V*_mem_ over a period of 10 μs in BE-RST-based LIF neuron. The amplitude of the *I*_in_ pulses is 5 μA. The *t*_int_ and pulse width (*t*_pulse_) are 1 μs and 50 ns, respectively. In the leaky-integration step, one *I*_in_ pulse increases the *V*_mem_ by 0.25 V while the *V*_out_ remains close to 0 V. This is because the increased *V*_mem_ is mostly applied to the high-resistance BE-RST rather than the *R*_out_. When the three *I*_in_ pulses are integrated, *V*_mem_ reaches the *V*_th_ of 1.01 V, and then the *V*_out_ increases sharply to 0.32 V. When the *V*_mem_ decreases to the *V*_rest_ of 0.26 V, the *V*_out_ returns to 0 V. This series of process completes the one cycle of spike generation in 3.3 μs. In particular, our proposed neuron exhibits low spiking energy consumption due to the low *V*_mem_ operating range and steep switching time induced by the heterojunction structure of BE-RST. This will be analyzed in detail through comparison with the RST of the homojunction structure in Fig. 3.

### Comparison between pure Si-RST and BE-RST based LIF neuron

Figure 3 compares the periodic spike responses based on the *V*_mem_ and *I*_A_ of pure Si-RST and BE-RST based LIF neurons over a period of 150 μs. The pure Si-RST features a (Si-based) homogeneous bandgap structure and is otherwise identical to BE-RST. The amplitude of the *I*_in_ pulses is 1 μA. The *t*_int_ and *t*_pulse_ are 10 μs and 200 ns, respectively. Over the preceding 200 μs duration, the periodic spiking operation is reliably iterated through the integration of *I*_in_ pulses. In the pure Si-RST neuron, the *V*_out_ of 0.26 V is generated when the *V*_mem_ increases to *V*_th_ of 2.1 V, as shown in Fig. 3a. Meanwhile, in the proposed neuron, the *V*_out_ of 0.37 V is achieved when the *V*_mem_ reaches to the low *V*_th_ of 1.1 V, as shown in Fig. 3b. Moreover, the on–off switching time in BE-RST neuron is 0.3 μs, which is 14 times shorter compared to 4.2 μs in pure Si-RST neuron. The low *V*_th_ and short on–off switching time can result in low energy consumption per spike (*E*_spike_). The *E*_spike_ of BE-RST neuron is compared to pure Si RST neuron using the following Eq. (1).

1 $$ E_{spike} = \int_{T} {V_{mem} (t)} \cdot I_{A} (t)dt $$

Here, *T* includes the time it takes RST to remain the on-state as well as the time it takes to return completely to the off-state. The pure Si-RST neuron consumes 2.08 pJ of *E*_spike_. In contrast, the BE-RST neuron consumes 0.36 pJ of *E*_spike_, which is 5.8 times more energy efficient than the pure Si-RST neuron.

**Fig. 4:**
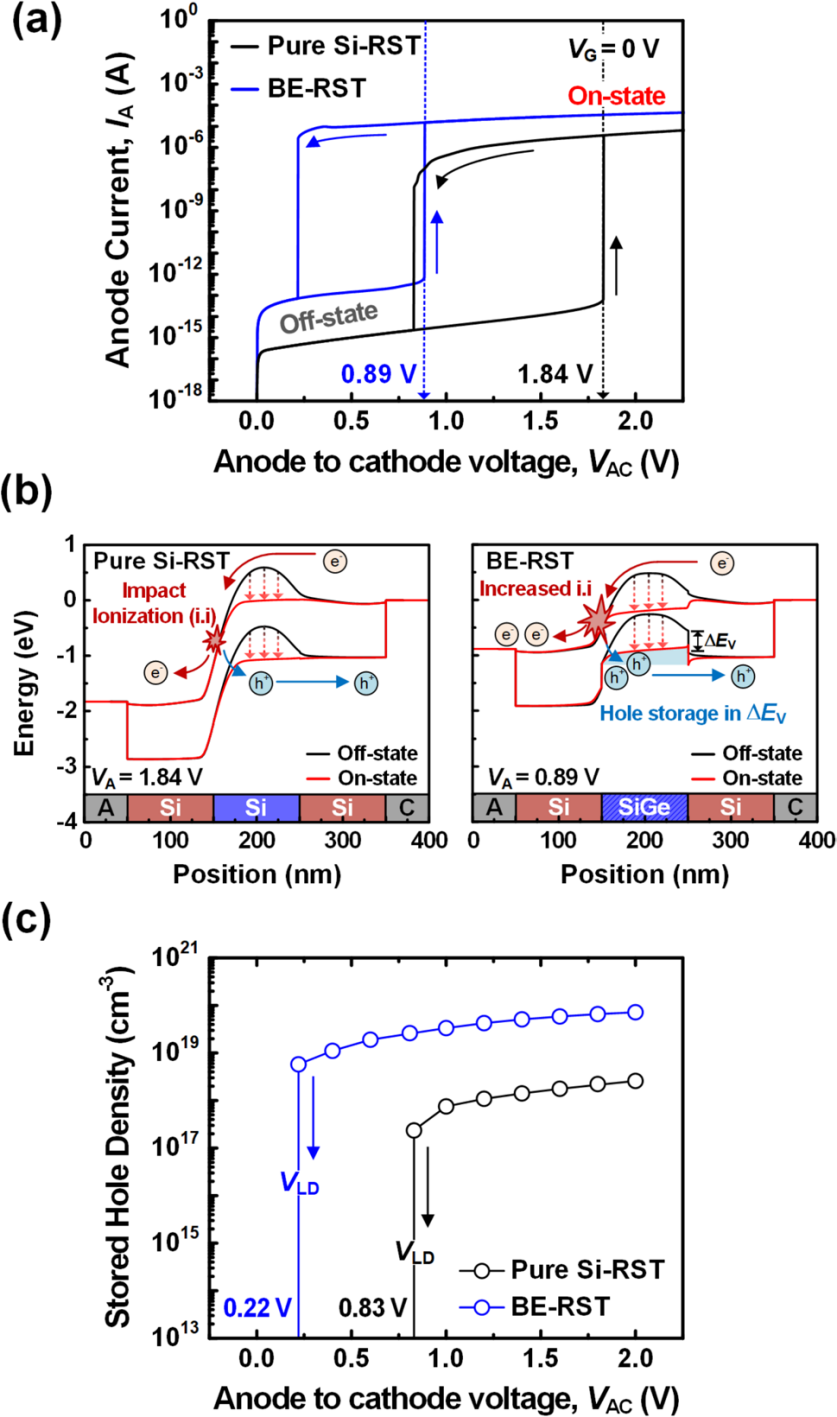
**a** *I*_A_-*V*_AC_ hysteresis characteristics of the pure Si-RST and BE-RST, **b** energy band of the pure Si-RST and the BE-RST when the latch-up phenomenon initiates and completes and **c** stored hole density of the pure Si-RST and the BE-RST

Figure 4a compares the hysteresis characteristics between the pure Si-RST and BE-RST to analyze the low *E*_spike_ of BE-RST neuron in detail. The low *E*_spike_ of BE-RST neuron is due to two main factors: low *V*_th_ and short on–off switching time. First, the low *V*_th_ is determined by the low *V*_LU_. The *V*_LU_ of BE-RST is 0.89 V, which is 2.1 times lower than 1.83 V of pure Si-RST. Figure 4b shows energy band of the pure Si-RST and BE-RST when the latch-up phenomenon initiates and completes, to understand the low *V*_LU_ of BE-RST. At the *V*_LU_, the excess holes are sufficiently supplied by impact ionization as the positive *V*_AC_ moves electrons from the cathode region into the *p*-channel. The excess holes accumulate in the *p*-channel, reducing the potential barrier. This further promotes the injection of more electrons into the *p*-channel and generation of electron–hole pair. As this process occurs iteratively, it activates positive feedback within the *p*-channel, ultimately transitioning RST to the on-state. In other words, it can be considered that the low *V*_LU_ is determined by the more efficient supply and storage of excess holes in the *p*-channel. In the BE-RST, the narrow *p*-channel bandgap increases the impact ionization coefficient, leading to the effective supply of excess holes. Furthermore, the valence bandgap offset (∆*E*_v_) suppresses the diffusion of stored excess holes, enabling efficient storage of excess holes [37–39]. Next, the short on–off switching time is explained by the fast discharging induced through high on-current (*I*_on_) level. Here, the short on–off switching time is described by fast discharging induced through high on-current (*I*_on_) level. As shown in Fig. 4a, the *I*_on_ level of pure-Si RST considerably decays from 3.7 μA to 11 nA during reverse *V*_AC_ sweeping. In comparison, the BE-RST maintains a high *I*_on_ level from 15 μA to 2 μA. The *I*_on_ level is influenced by the impact ionization coefficient and hole storage capability. The crucial point here is that the BE-RST operates within a low voltage range. In the low voltage range, the hole storage capability has a greater influence on the BE-RST than the impact ionization coefficient [37]. The reason is that the impact ionization coefficient is especially amplified at high voltages, while the hole storage capability can effectively reduce the potential barrier even at low voltages. Figure 4c shows the high stored hole density inside the *p*-channel of BE-RST compared to the pure Si-RST in the on-state. The pure-Si RST stores 2.2 × 10^17^ cm^−3^ holes at the *V*_LD_ of 0.83 V. In contrast, the BE-RST stores 5.7 × 10^18^ cm^−3^ holes even at the lower *V*_LD_ of 0.22 V. The BE-RST can keep the potential barrier smaller by efficiently storing excess holes through the valence bandgap offset described above, thus retaining large *I*_on_ until it latches down.

### Tunable function

**Fig. 5:**
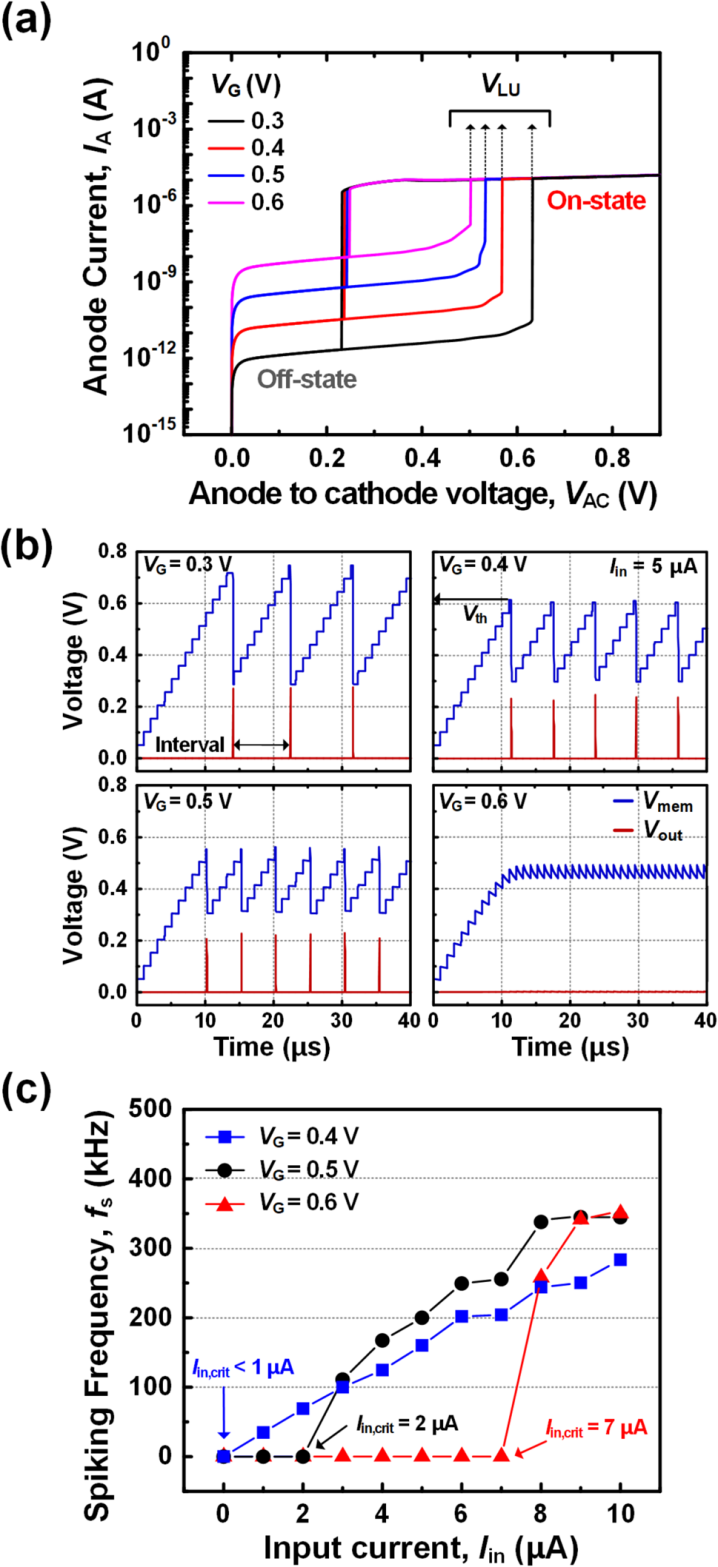
**a** *I*_A_-*V*_AC_ hysteresis characteristics at various *V*_G_, **b** *V*_mem_ and *V*_out_ in time domain at various *V*_G_ and **c** *f*_s_ according to the *I*_in_ at the various *V*_G_

Figure 5a shows the *I*_A_-*V*_AC_ hysteresis characteristics according to the modulation of the *V*_G_. As the* V*_G_ increases from 0.3 V to 0.6 V, the off-current increases from 22 pA to 156 nA, while the *V*_LU_ decreases from 0.63 V to 0.51 V. The larger *V*_G_ lowers the potential barrier, allowing more electrons that make up the off-current to enter the *p*-channel. The increase in injected electrons amplifies impact-ionization rate, activating the positive feedback at smaller *V*_AC_.

Figure 5b shows the periodic spike response that can be tuned by varying *V*_G_ values. As the *V*_G_ increases from 0.3 V to 0.5 V, the interval between spikes decreases from 9 µs to 5 µs, while the *V*_out_ decreases from 0.28 V to 0.22 V. The reduced interval is attributed to the shorter integration time as *V*_th_ decreases. The *V*_out_ is also determined by the *V*_th_. Here, the ratio between *V*_out_ and *V*_th_ is constant because the on-state current level remains almost constant regardless of the *V*_G_. In other words, the *V*_th_ is distributed to BE-RST and *R*_out_ in a constant ratio. It is worth noting that no spike event occurs at *V*_G_ = 0.6 V as the *V*_mem_ fails to reach the *V*_th_ of 0.51 V. This means that the amount of charge leaked due to the off-current during the *t*_int_ (*Q*_leak_) is larger than the amount charged to the *C*_mem_ per *t*_pulse_ (*Q*_mem_). That is, spikes can be generated when the *I*_in_ is larger than critical *I*_in_ value (*I*_in,crit_) that satisfies the condition *Q*_mem_ = *Q*_leak_ at the *V*_th_. Therefore, when the *V*_G_ is above 0.6 V, the spike generation can be suppressed by inducing active leaky operation with high off-current.

Figure 5c shows the spiking frequency (*f*_s_) as a function of the *I*_in_ at *V*_G_ = 0.4 V, 0.5 V and 0.6 V. The *I*_in_ pulse has the *t*_pulse_ of 10 ns and the *t*_int_ of 1 μs. When the *I*_in_ increases, the *f*_s_ increases due to the faster charging speed. The larger *V*_G_ increases the increasing rate of the *f*_s_ with respect to the *I*_in_. When the larger *V*_G_ is applied, the interval between spikes reduces due to the lowered *V*_th_, resulting in a significant increase in the *f*_s_. Moreover, it is noteworthy that the *I*_in,crit_ shows a more rapid increase as *V*_G_ increases. This is due to the significant increase in the *Q*_leak_. The *Q*_leak_ becomes very large as the thermionic emission rises exponentially with the reduction of the potential barrier. This modulation of the *I*_in,crit_ enables selective response to certain range of input, inducing an energy efficient sparse activity and high learning accuracy [25, 27].

**Fig. 6:**
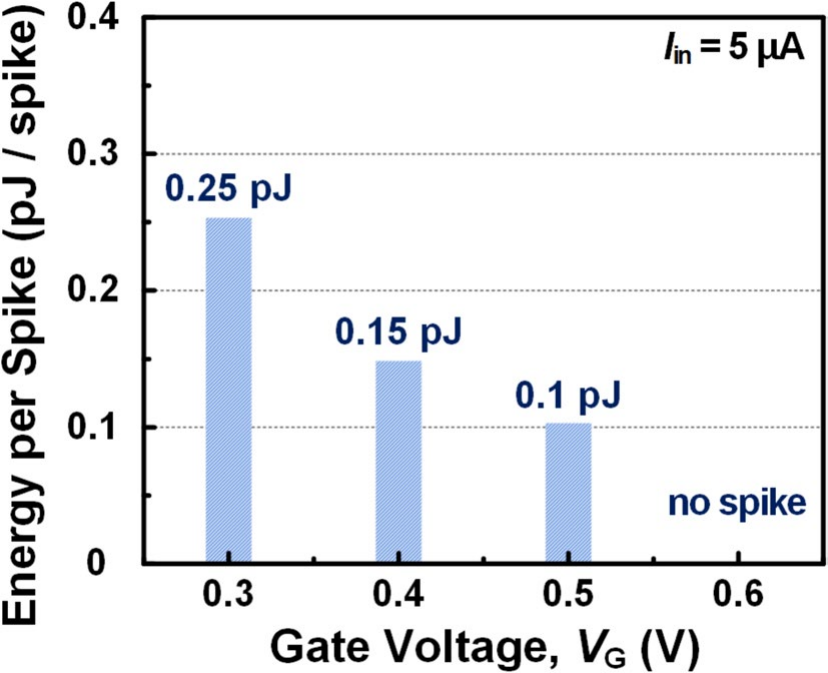
The effects of the *V*_G_ on *E*_spike_

Figure 6 shows the effect of adjusting the *V*_G_ on the *E*_spike_ when the *I*_in_ is 5 μA. The *E*_spike_ decreases linearly from 0.25 pJ to 0.10 pJ as the *V*_G_ increases from 0.3 V to 0.5 V, except for *V*_G_ = 0.6 V where the *I*_in,crit_ is larger than *I*_in_ = 5 μA. This is mainly due to the decrease in the *V*_LU_, which determines the *V*_th_. The formula for *E*_spike_ described above can be simplified to the product of the time BE-RST remains on-state, the *I*_on_ level, and *V*_th_. As the *V*_G_ increases, only *V*_th_ decreases, while the *I*_on_ level maintains nearly constant with little difference in the on–off transition time. Therefore, the *E*_spike_ can be fine-tuned through predictions taking into account the tendency of *V*_th_ with respect to *V*_G_.

### Performance comparison with other LIF neurons

In Table 1, BE-RST LIF neuron is compared with other LIF neurons in terms of core device, input & output type, energy per spike, tunability, spiking frequency and external circuit. The CMOS circuit-based neurons are composed of numerous components for neuronal functions, resulting in low density and large energy consumption [7]. To overcome these limitations, various core devices have been utilized in LIF neuron. The PD SOI-MOSFET has a wide spiking frequency range (~ 20 MHz), but consumes a lot of energy (~ 35 pJ) [9]. The L-BIMOS and DG-JLFET neuron achieve low energy consumption (~ 0.18 pJ) with a very wide frequency range (~ 2.3 GHz) [10, 11]. The wide frequency range of the PD SOI-MOSFET, L-BIMOS and DG-JLFET neuron is due to their integration mechanism based on floating-body effect. However, significant energy can be additionally consumed in these floating-body Si neurons which require external circuit for voltage to current signal conversion and reset operation. Single MOSFET neuron can improve the homeostasis of neural system with its tunable threshold voltage but requires large energy for spiking behavior [15]. NbOx TS-device neuron and Ag/HfO_2_ TS-device neuron can realize low-energy neural oscillation without external circuit but, their gate-less structure does not provide tunable functions [18, 19]. On the other hand, our BE-RST neuron does not require any external circuits and can achieve a sufficiently wide tunable spiking frequency range with low-energy consumption.

**Table 1:** Performance comparison of the proposed neuron with other neurons

Ref.	Core device	In → Out	Energy/ Spike	Tunability	Spiking Frequency	External circuit
[7]	CMOS	*I* → *V*	960 pJ	No	~ 100 kHz	No
[9]	PD SOI-MOSFET	*V* → *I*	35 pJ*	No	~ 20 MHz	Yes
[10]	L-BIMOS	*V* → *I*	0.18 pJ*	No	~ 2.3 GHz	Yes
[11]	DG-JLFET	*V* → *I*	1.14 pJ*	No	~ 1 MHz	Yes
[15]	MOSFET	*I* → *V*	950 pJ	Yes	~ 500 Hz	No
[18]	Ag/HfO_2_-TS dev. (*C*_mem_ = 1pF)	*V* → *I*	0.29 pJ	No	-	Yes
[19]	NbOx-TS dev. (*C*_mem_ = 1pF)	*V* → *I*	2 pJ	No	~ 225 kHz	No
This work	BE-RST	*I* → *V*	0.36 pJ	Yes	~ 350 kHz	No

*Energy consumption of external circuits is excluded

## Conclusion

In this work, we have demonstrated a low-energy and tunable LIF operation based on the hysteresis characteristics of gated BE-RST. The BE-RST with heterojunction structure exhibits 4 times amplified *I*_on_ at 2.1 times reduced *V*_LU_ compared to pure Si-RST thanks to its improved impact ionization and robust hole storage capability. Due to its hysteresis characteristics, the proposed LIF neuron consumes 0.36 pJ of energy per spike, which is 5.8 times lower than 2.08 pJ/spike of the pure Si-RST neuron. Moreover, gate bias modulates the *V*_LU_ and the off-current level, which controls the input response range and spiking frequency. These tunable properties at neuron level can enhance energy efficiency and learning accuracy without hardware modification. Therefore, our proposed area- and energy-efficient neurons can be a promising candidate to play a key role in the SNN implementation. Our future work will focus on comprehensively evaluating the practical performance range of the proposed neuron, including assessments of manufacturing variability and device durability. Additionally, the SNN simulations will be conducted to demonstrate the benefits of the tunable threshold function.

## Simulation methods

**Fig. 7:**
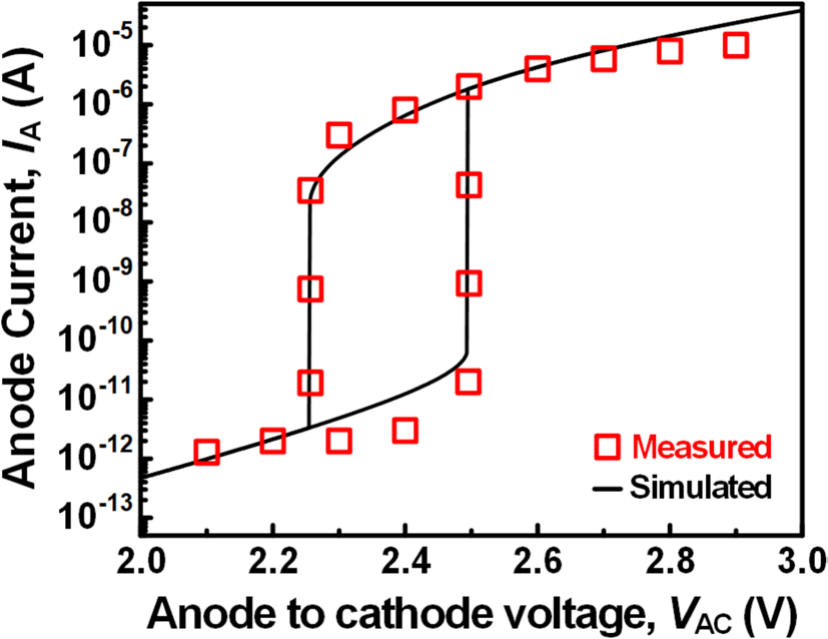
*I*_A_-*V*_AC_ hysteresis characteristics simulation results (black line) calibrated to the experimental data (red symbol) of Si nanowire *biristor* device [41, 42]

The device characteristics of RST (pure-Si RST and BE-RST) and LIF operation of the proposed neuron were all simulated using TCAD sentaurus tool [40]. The basic physical models used in the simulations include Fermi–Dirac distribution, Drift–diffusion transport, Philips unified mobility, High-field saturation, Doping-dependent mobility, Avalanche generation, Doping-dependent Shockely-Read-Hall (SRH), Auger recombination models. In addition, SRH recombination model for Si-SiGe surface was also applied to consider hetero-junction defects in the BE-RST. As shown in Fig. 7, the parameters of physical models were well calibrated to match the experimental data from *I*_A_- *V*_AC_ characteristic curve of the Si nanowire *biristor* device, that is most similar to RST. Both devices consist of physical nanowire *n*^+^-*p*-*n*^+^ layers and exhibit hysteresis characteristics through the impact ionization effect [41, 42].

## Data Availability

The data generated and/or analyzed during the current study are not publicly available for legal/ethical reasons but are available from the corresponding author on reasonable request.
